# Practical Notes on Diagnosis and Treatment

**Published:** 1908-03-28

**Authors:** 


### Practical Notes on Diagnosis and Treatment.

**Boric Starch Poultice.**

Mix a teaspoonful of boric acid and a tablespoonful of starch to a paste with cold water, then add a pint of boiling water. Spread on cotton covered with muslin and change often. Used for removing the crusts in eczema and impetigo contagiosa, etc.

**Optic Neuritis in Intracranial Tumour.**

I find that out of 50 cases the neuritis in 19 was more intense in the eye on the same side as the lesion; in 11 it was more marked in the opposite eye; in 13 it was equal on the two sides, or there was no note to say there was any difference; and in seven cases there was no neuritis.

**Turpentine Vapour in Bronchitis.**

In cases of bronchitis with tenacious secretion which the patient is unable to expel, so that cyanosis develops, the inhalation of turpentine vapour may be very useful. An ounce of oil of turpentine is added to four ounces of boiling water in an inhaler, and the patient is encouraged to inhale as freely as possible.

**Salicylates and Hæmaturia.**

Some cases have been reported in which the administration of sodium salicylate to children has been followed by hæmaturia.

**Venesection in Apoplexy.**

Venesection in apoplexy is warranted by a full, tense pulse, a labouring heart, with failure and acute distension of the right auricle and ventricle, and engorgement of the cervical veins.—*Dr. Leonard Guthrie.*

**Nocturnal Enuresis.**

Many failures in treatment are due to the use of belladonna in too small doses. At any period beyond infancy 5 minims of the tincture should only be considered an initial dose; in a child of five years it is usually safe to commence with 10 minims. Then every fifth or sixth day this dose should be increased by 2½ minims until either the enuresis is stopped or the limit of tolerance is reached.

